# Innate Immune Sensors and Cell Death—Frontiers Coordinating Homeostasis, Immunity, and Inflammation in Skin

**DOI:** 10.3390/v17020241

**Published:** 2025-02-10

**Authors:** Ye Mon Soe, Seen Ling Sim, Snehlata Kumari

**Affiliations:** Frazer Institute, The University of Queensland, Dermatology Research Centre, Woolloongabba, Brisbane, QLD 4102, Australia; y.soe@uq.edu.au (Y.M.S.); s.sim@uq.edu.au (S.L.S.)

**Keywords:** skin homeostasis, innate immune sensors, programmed cell death, apoptosis, necroptosis, pyroptosis, PANoptosis, inflammation, NF-κB

## Abstract

The skin provides a life-sustaining interface between the body and the external environment. A dynamic communication among immune and non-immune cells in the skin is essential to ensure body homeostasis. Dysregulated cellular communication can lead to the manifestation of inflammatory skin conditions. In this review, we will focus on the following two key frontiers in the skin: innate immune sensors and cell death, as well as their cellular crosstalk in the context of skin homeostasis and inflammation. This review will highlight the recent advancements and mechanisms of how these pathways integrate signals and orchestrate skin immunity, focusing on inflammatory skin diseases and skin infections in mice and humans.

## 1. Skin Homeostasis and Immunity

### 1.1. Skin Structure

**Figure 1:**
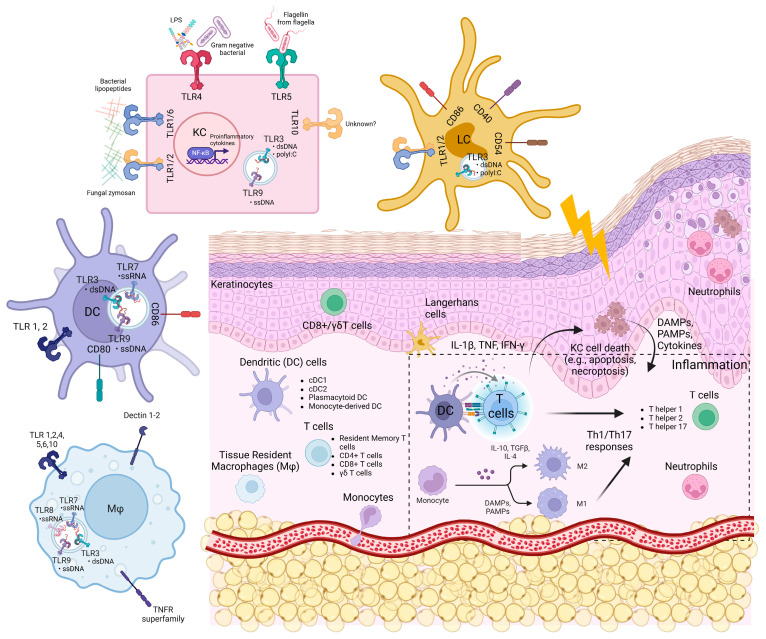
The skin structure, skin cells, and expression of innate immune sensors.

The skin consists of the epidermis, the dermis, and the hypodermis. The epidermis is the outermost layer of the skin. It mainly contains epithelial cells called keratinocytes, as well as other immune and non-immune cells such as melanocytes, Langerhans cells (LCs), and CD8^+^ T cells [1,2,3]. The mouse epidermis also harbours dendritic epidermal T cells (DETCs), which are absent in humans [4]. The dermis lies below the epidermis and harbours diverse groups of immune cells such as dendritic cells (dermal DCs, plasmacytoid DCs), mast cells, macrophages, T-lymphocytes (CD4^+^ T helper 1 (T_H_1), T_H_2, T_H_17 cells, γδ T cells), natural killer cells, and fibroblasts. They are integrated within a framework of extracellular matrices (collagen, elastic tissues, and reticular fibers) and lymphatic and blood vessels (Figure 1) [1,2,3].

**Figure 2:**
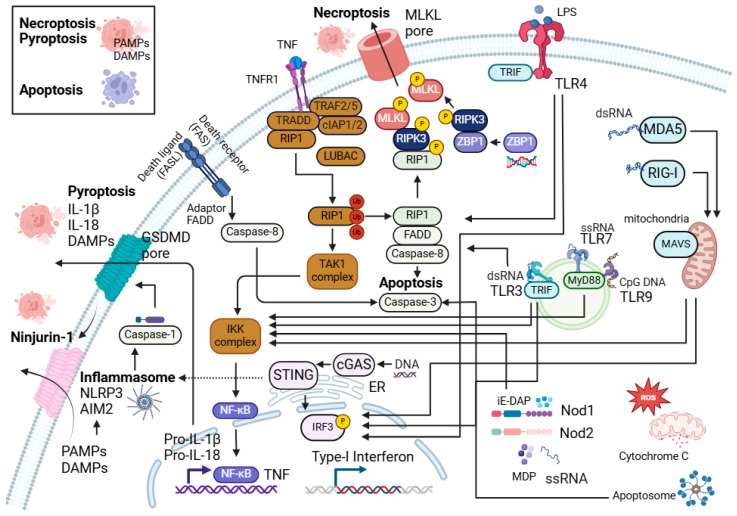
Programmed cell death, innate immune signalling, and their interactions. Interaction between the programmed cell death (PCD) pathways and NF-κB signalling central to the innate immune sensors in the skin cells. Different innate immune sensors such as TLRs, NLRs, nucleic acid sensors (AIM-2, cGAS-STING, RIG-I, MDA-5, and ZBP1), as well as cell death can directly or indirectly promote inflammatory responses and inflammation in the skin.

Keratinocytes and innate immune cells are the key sentinels in the skin. They express pattern recognition receptors such as toll-like receptors (TLRs), NOD-like receptors (NLRs), RIG-I-like receptors (RLRs), and nucleic acid sensors, which enable them to sense foreign pathogens and molecules such as PAMPs (pathogen-associated molecular patterns) and host DAMPs (damage-associated molecular patterns) (Figure 1 and Figure 2). They also possess molecular frameworks facilitating programmed cell death, such as apoptosis, necroptosis, and pyroptosis (Figure 2) [5,6,7].

Epithelial–immune cell communication is essential for skin homeostasis [3,8,9]. The dysregulation of epithelial–immune cell communication can lead to the development of inflammatory skin conditions [3,10,11].

### 1.2. Skin Immunity

Skin homeostasis is maintained through a coordinated network of immune cells, non-immune cells, effector molecules (e.g., cytokines, chemokines), and immunomodulatory signalling pathways (e.g., NF-κB, AP-1). Innate immune cells, such as myeloid cells and adaptive immune cells, mainly lymphoid cells, are the major immune cell types in the skin. Both cellular pools are derived from the hematopoietic stem cell lineage [12].

Myeloid cells in the skin include DCs, LCs, macrophages, monocytes, and neutrophils. These cells actively engage in functions such as phagocytosis, antigen-presentation, antimicrobial peptide production, cytotoxic granules, cytokines and chemokines secretion, and provide an immediate response to stimuli. They also clear dead cells and tissue debris and promote tissue repair. These cells also coordinate the activation of the adaptive arm of skin immunity.

Lymphoid cells in the skin include CD4^+^ T helper cells, CD8^+^ cytotoxic T cells, regulatory T cells, resident memory T cells, gamma delta T cells (γδ T cells), and B lymphocytes. They are responsible for targeting specific immune responses and promoting long-term and rapid immunity upon the recurrence of diseases and infections [13].

Collectively, both myeloid and lymphoid immune cells facilitate a balanced and dynamic immune environment to respond to inflammatory stimuli and restore and maintain skin homeostasis.

Tightly regulated epithelial–immune signalling crosstalk is essential to maintain skin homeostasis. Keratinocytes and innate immune cells (e.g., macrophages, Langerhans cells, and dendritic cells) harbour several innate immune sensors (e.g., TLRs, NLRP inflammasomes, etc.) that help to mount immune responses. Dysregulated innate immune sensing in the skin is linked to cell death (e.g., pyroptosis and necroptosis) and autoimmune/inflammatory skin diseases (e.g., psoriasis and cutaneous lupus). DAMPs and PAMPs released from dead cells, particularly those from pyroptosis and necroptosis, can further amplify the responses of innate immune sensors in a positive feedback loop, exacerbating inflammation and death in the skin in unison with the production of pro-inflammatory cytokines such as TNF, IL-1β, IFN-γ, and others.

## 2. NF-κB, Programmed Cell Death, and Skin Immunity

Programmed cell death plays a key role in maintaining skin immunity [14,15,16,17]. Apoptosis, necroptosis, and pyroptosis are the most characterised types of programmed cell death. Figure 2 highlights these forms of cell death, their components, and interactions. Apoptosis is widely recognised as a non-inflammatory and immunologically silent form of cell death, where cellular components are enclosed in apoptotic bodies [18]. However, excessive apoptosis can drive inflammation in the skin [19,20,21,22,23]. In contrast to apoptosis, necroptosis, and pyroptosis are inflammatory forms of programmed cell death, in which the cells rupture and disrupt their plasma membrane integrity [14,15,16]. This occurs through the formation of mixed lineage kinase domain-like (MLKL) pores in necroptosis [24] and Gasdermin D pores in pyroptosis [25], resulting in the release of endogenous DAMPs such as high mobility group box 1 (HMGB-1) [26,27,28,29], heat shock proteins (HSPs) [30], and nucleic acids [31,32] thus exacerbating inflammation. A membrane protein, Ninjurin-1 (NINJ1), has also been found to be involved in plasma membrane rupture and the release of DAMPs in lytic cell death, including pyroptosis and necroptosis, hence promoting inflammation [33].

The relationship between programmed cell death and inflammation is a double-edged sword. Inflammation is essential to the mounting of immune responses during infections or tissue injuries, but it must also be controlled and resolved to restore skin homeostasis. If the interplay between cell death and inflammation is not properly regulated, this could drive excessive and persistent inflammation, which may contribute to the pathogenesis of chronic inflammatory skin diseases.

Programmed cell death has been associated with several inflammatory skin diseases such as psoriasis [34] and toxic epidermal necrolysis [35]. However, their relevance to skin diseases is not fully understood. Previous studies in mouse models have shown that dysregulated NF-κB signalling and programmed cell death in the keratinocytes drive skin inflammation [14,17,36,37]. Both the overactivation and inhibition of NF-κB in the epithelial cells of the skin led to the development of inflammatory skin phenotypes in mice [17,38]. Furthermore, mice with dysregulated programmed cell death such as the epidermal keratinocyte-specific deletion of apoptotic components—Fas-associated protein with death domain (FADD^E-KO^) [39] or caspase-8 (Caspase-8^E-KO^) [40,41]—or apoptotic/necroptotic components—receptor-interacting protein kinases (RIPKs) 1 (RIPK1^E-KO^) [42]—experienced the development of inflammatory skin lesions. The skin phenotype of FADD^E-KO^ and RIPK1^E-KO^ mice were characterised by epidermal hyperplasia, the upregulation of cytokines/chemokines, and the accumulation of immune cells and was driven by necroptosis. Crossing FADD^E-KO^ or RIPK1^E-KO^ mice with *Ripk3* or *Mlkl* knockout mice prevented the development of skin lesions [39,42,43].

An overactivation of NF-κB in mice by deleting NF-κB inhibitor alpha (IκBα), a cytoplasmic inhibitor of NF-κB, resulted in a T cell-mediated severe skin inflammatory phenotype, leading to lethality at postnatal day (P) 8 [44]. Moreover, an overexpression of the inhibitor of NF-κB kinase (IKK2), a kinase activating NF-κB, in the epithelial cells also led to the development of inflammatory skin phenotype and the recruitment of macrophages and T cells in the skin by P14 [45].

The inhibition of NF-κB or the dysregulation of the NF-κB pathway components also led to the development of inflammatory skin disease. Keratinocyte-specific deletion of the gene encoding for the NF-κB essential modulator (NEMO), a regulatory subunit of the IKK complex in the NF-κB pathway, led to the development of inflammatory skin lesions at P2 and lethality at P6 [46]. The skin lesion development of the epidermis-specific NEMO knockout mice (NEMO^E-KO^) was partially dependent on TNFR1 signalling, as the genetic inhibition of TNFR1 prevented the development of skin lesions in these mice at an early stage [46]. However, the NEMO^E-KO^/*Tnfr1*^−/−^ mice developed skin inflammation in adulthood [46]. NEMO deficiency in humans also causes a genetic disorder, incontinentia pigmenti, which is characterised by the development of skin lesions, as well as other complications, and is detrimental to males [47]. An overexpression of the degradation-resistant super--repressor of IκBα (K5-IκBαSR and K14-IκBαM) caused inflammation [48,49]. K5-IκBαSR mice developed squamous cell carcinoma in adulthood in a TNF-dependent manner [48,50], where the K14-IκBαM mice died by P5–P7. The inhibition of IKK2 in the keratinocytes resulted in microscopic inflammatory skin lesions at P3–P4, which reached severity and led to lethality at P7–P9 [51,52]. Crossing IKK2^E-KO^ mice with full-body or epithelial cell-specific TNFR1-deficient mice rescued the skin lesion development, showing that TNFR1 signalling in keratinocytes drives the development of the inflammatory skin phenotype in these mice [51,52,53]. In subsequent studies, interleukin (IL)-24 signalling and cell death were identified as major drivers of the inflammatory skin phenotype of the IKK2^E-KO^ mice [52,53]. The genetic inhibition of the components of necroptosis, RIPK3 or mixed lineage kinase domain-like pseudokinase (MLKL) (IKK2^E-KO^/*Ripk3*^−/−^ or IKK2^E-KO^/*Mlkl*^−/−^), significantly ameliorated the severity of the skin disease [52], and the combined inhibition of both the necroptotic and apoptotic pathways by the global deletion of RIPK3 and FADD (IKK2^E-KO^/FADD^E-KO^/*Ripk3*^−/−^) completely prevented the inflammatory skin phenotype development in these mice [52]. Lastly, the deletion of both NF-κB subunits, RelA and c-Rel, in keratinocytes (RelA^E-KO^/cRel^E-KO^) led to the development of inflammatory skin lesions similar to the IKK2^E-KO^ mice [52,54]. Crossing them with RIPK1 kinase mutated mice (Ripk1^D138N/D138N^) or *Mlkl*^−/−^ mice prevented the inflammatory skin phenotypes observed in RelA^E-KO^/cRel^E-KO^ mice at an early age, and they only developed mild lesions between the age 6–9 months or 3–4 months, respectively [52]. These data demonstrated that NF-κB acts as a checkpoint regulator to prevent epithelial cell death, and the inhibition of NF-κB signalling results in cell death and inflammation in the skin.

The cellular inhibitor of apoptosis protein (cIAP1) is a component of the NF-κB pathway [55,56,57,58]. Studies have shown that the inhibition of cIAP1 in the epidermis, as well as cIAP2, ubiquitously leads to skin inflammation and cell death [19]. Additionally, the deletion of tumour necrosis factor receptor-associated factor 2 (TRAF2) in keratinocytes (TRAF2^E-KO^) leads to a TNF-dependent epidermal thickening and skin inflammation [20]. The deletion of MLKL did not prevent the onset of skin inflammation in the TARF2^E-KO^ mice [20]. However, the simultaneous deletion of MLKL and caspase-8 rescued the skin phenotype, suggesting that apoptosis, and not necroptosis, led to the inflammatory skin phenotype in these mice [20].

The global mutation of *cpdm* (encodes for SHANK-associated RH domain interactor (SHARPIN)), a component of the linear ubiquitin chain assembly complex (LUBAC), resulted in the spontaneous development of inflammatory skin lesions (*Sharpin^cpdm^*) [21,22,23]. LUBAC consists of haem-oxidised iron-responsive element-binding protein 2 (IRP2), ubiquitin ligase-1 (HOIL-1), HOIL-1-interacting protein (HOIP), and SHARPIN, which adds linear ubiquitin to NF-κB signalling components such as NEMO to promote the nuclear translocation of NF-κB [59]. Further studies on this model showed the prevalence of massive cell death in the epidermis of *Sharpin^cpdm^* mice. Moreover, the skin inflammation in these mice was driven by TNF, epithelial TNFR1 and RIPK1 kinases, FADD/ caspase-8-mediated cell death, and innate immune sensors, adaptors and components of inflammasomes [22,23,60,61,62,63,64,65].

Deficiency of the linear ubiquitin chain-specific deubiquitinase, OTULIN, causes OTULIN-related autoinflammatory syndrome (ORAS) in humans. Mice lacking Otulin (OTULIN^E-KO^) also develop an inflammatory skin disease [66,67]. Similar to *Sharpin^cpdm^* mice, the phenotype of OTULIN^E-KO^ mice is driven by TNFR1 and RIPK1 kinases. Moreover, RIPK3 or MLKL deficiency ameliorates skin lesion development, while the combined deletion of epidermal FADD and RIPK3 prevents skin lesion development [66,67].

In summary, these studies have shown that dysregulated NF-κB signalling and the programmed cell death pathway initiate the inflammatory skin phenotype in mice; their crucial roles in skin immunity have also been highlighted.

## 3. Innate Immune Sensors and Cell Death: Expression, Activation Signalling, and Their Contribution to Skin Inflammation and Immunity

The skin cells harbour several innate immune sensors that serve as the first line of defence to detect PAMPs and DAMPs. These sensors include TLRs, C-type lectin receptors, cytoplasmic sensors such as NOD-like receptors (NLRs), nucleic acid sensors such as RLRs, cyclic guanosine monophosphoate (GMP)–adenosine monophosphate (AMP) synthase-stimulator of interferon genes (cGAS-STING), interferon gamma-inducible protein 16 (IFI16), Z-DNA binding protein 1 (ZBP1), and absent in melanoma 2 (AIM2) [68,69,70,71]. In the skin, these pattern recognition receptors (PRRs) work in unison to mount immune responses and maintain skin homeostasis. Figure 1 shows the expression of these innate immune sensors on various cell types in the skin, and Figure 2 shows their downstream signalling and interactions with cell death pathways.

### 3.1. Toll-like Receptors (TLRs)

TLRs recognise the conserved molecular patterns of microbes, known as PAMPs, and promote the recruitment and activation of innate immune cells such as macrophages and DCs, which are essential to the coordination of innate and adaptive immunity. TLRs are type I integral glycoproteins with three domains—an N-terminus ligand recognition domain, a transmembrane domain, and a C-terminus cytoplasmic signalling domain. The recognition of microbial components and host stimuli through TLRs activates downstream signalling cascades, such as NF-κB and mitogen-activated protein kinase (MAP) kinases, resulting in the expression of cytokines and chemokines [72,73,74].

TLRs are grouped into two different categories based on their localisation. TLR1, TLR2, TLR4, TLR5, TLR6, and TLR10 are localised at the cell surface membrane to sense microbial cell wall components, while TLR3, TLR7, TLR8, and TLR9 mainly detect nucleic acid components and are localised at the endosome of the cells [72,73,75]. Myeloid differentiation primary response 88 (MyD88) and toll/interleukin-1 receptor (TIR)-domain-containing adapter-inducing interferon-β (TRIF) are the two adaptor molecules in the TLR pathways. All TLRs can utilise MyD88-dependent signalling except TLR3. This initiates the formation of a complex consisting of IL1R-associated kinase (IRAK) family kinases such as IRAK1, IRAK3, IRAK4, and IRAK-M [72,76,77,78]. This leads to the recruitment of tumour necrosis factor receptor-associated factor 6 (TRAF6), which activates transforming growth factor beta-activated kinase 1 (TAK1) to phosphorylate the IKK complex. IKK2 then phosphorylates IκBα, resulting in the proteasome-mediated degradation of IκBα and the dissociation of NF-κB to translocate to the nucleus for the activation of the NF-κB-mediated genes. TAK1 can also activate the MAPK pathway to allow for the translocation of AP-1 to the nucleus to stimulate the pro-inflammatory factors [72,76,77,78].

TLR3 and TLR4 use TRIF to recruit TRAF6 and/or TRAF3 [76,77,78]. TRAF6 recruits RIPK1 to activate TAK1 for the activation of NF-κB and MAPK pathways. Meanwhile, TRAF3 recruits TANK-binding kinase 1 (TBK1) and inhibitor of κB kinase ϵ (IKKϵ), which phosphorylate and translocate interferon regulatory factor 3 (IRF3) and eventually lead to the production of interferon-β (IFNβ) [76,77,78].

Epidermal keratinocytes constitutively express TLR 1–6, 9, and 10 and play an important role in cutaneous inflammation and antiviral responses [9,79,80,81,82]. TLR1 can detect lipoteichoic acid, lipopeptides, and peptidoglycans from bacteria and zymosan from fungus and forms heterodimer with TLR2 or TLR6 [9,81,83]. TLR3 binds to dsRNA, which can be released from viruses and damaged host cells during cellular stress. A synthetic TLR3 ligand, polyinosinic–polycytidylic acid (polyI:C), has been shown to induce proinflammatory cytokines such as IL-6, IL-8, IL-1β, TNF, IFN-β, IL-23p19, IL-8, CCL5, and CCL20 in keratinocytes [84,85,86,87]. Additionally, TLR3 upregulates genes such as ATP-binding cassette, sub-family A, member 12 (ABCA12), glucocerebrosidase, acid sphinoglyelinase, and transglutaminase 1, which are involved in skin barrier recovery and wound healing in normal human epidermal keratinocytes (NHKs) [87].

TLR4 and TLR5 in keratinocytes are crucial players in the cutaneous defence against Gram-negative pathogens. TLR4 activation via lipopolysaccharide (LPS) stimulation regulates the proinflammatory cytokines and chemokines [79]. Cytokines like IFN-γ have been shown to increase the LPS-mediated expression of *TLR2* and *TLR4* genes in human keratinocytes in vitro [88]. TLR5 is expressed on the basal layer of the epidermis [89,90]. Stimulation of a primary air-lifted organotypic keratinocyte culture with bacterial flagellin and transforming growth factor (TGF)-α, a growth and differentiation factor important during wound healing and psoriasis, increased the level of IL-8 production in the keratinocytes [90]. Upon the engagement of TLR4 or TLR5 with the respective ligands, the NF-κB pathway was activated, which promoted the upregulation of IL-8, a chemoattractant for neutrophils and other immune cells to the site of infection and injury. TLR7 and 8 recognise single-stranded RNA but are not constitutively expressed in keratinocytes [79]. However, studies have shown that the imiquimod (TLR7/8 agonist) can induce NF-κB-mediated immune response in keratinocytes in the presence of calcium [80,91]. TLR9 binds to DNA with unmethylated CpG motifs released from damaged cells, bacteria, and viruses [92]. The stimulation of keratinocytes with CpG-oligodeoxynucleotides led to the production of CXCL8 and TNF in a concentration- and time-dependent manner through the phosphorylation of IκBα and the activation of NF-κB subunit p65 [79]. Moreover, the engagement of TLR9 can induce CXCL10 and IFNα in keratinocytes [79]. TLR10 is expressed in primary keratinocytes, but the PAMPs associated with the receptor in the skin have yet to be defined [80].

Immune cells in the skin also express a variety of TLRs (Figure 1). Human skin LCs express mRNA encoding TLR 1-3, TLR5, TLR6, and TLR10. LCs are highly responsive to TLR2 and TLR3 ligands, leading to IL-6, IL-8, and TNF-α production. However, poly(I:C)-stimulated LCs do not produce IFN-α/β [93,94]. In another study, poly(I:C) stimulation of LCs for two days led to the upregulation of co-stimulatory molecules (CD40, CD54, CD86), enhanced LC survival, and upregulated CXCL10, IL-6, and IL-12p40 [95]. LC-like DCs are derived from CD34^+^ progenitor cells and express comparable levels of TLR1-10 mRNA as monocyte-derived DCs [96]. They are responsive to TLR2 and TLR7/8 ligands, leading to the production of IL-8, IL-12, CCL3, CCL4, and TNF [96]. TLR3 stimulation of LC-like DCs also leads to IFN-β production, suggesting that LC might play a role in the antiviral activity within the skin [96]. Lastly, imiquimod and R-848 (TLR7/8 ligands)-treated human epidermal LCs promoted T cell proliferation in a primary mixed lymphocyte reaction, leading to T cell-mediated IFN-γ, IL-12, IL-1β, and TNF production [97].

Immature myeloid DCs express TLR 1–3, and their stimulation induces maturation as confirmed by the expression of co-stimulatory molecules, CD80 and CD86. The stimulation subsequently led to IL-12 production and the promotion of the Th1 immune response [98]. Plasmacytoid DCs express TLR7 and 9 [99,100], and the stimulation of TLR9 through CpG DNA induced type I IFN production [99,100]. Monocytes predominantly express *TLR1*, *TLR2*, *TLR4*, *TLR5*, and *TLR8* [99,100]. Engaging these TLRs has been shown to enhance the phagocytic ability of macrophages and the release of TNF and IL-6 [101].

Mast cells in the skin mediate IgE allergic reactions and express TLR 1–7 and 9 mRNA transcripts. The activation of TLR3 and TLR9 by poly(I:C) and CpG DNA, respectively, inhibited mast cell adhesion to vitronectin and fibronectin in vitro [102]. Moreover, poly(I:C) stimulation of the mast cells resulted in the production of type I IFN. These studies suggest that mast cells may have a role in antiviral immunity [103]. T cells in the skin also express TLRs 1–10s [104]. Stimulation of T cells via the CD3 monoclonal antibody and TLRs 2, 5, 8, and 9 ligands leads to T cell proliferation and IFN-γ production [105,106,107,108,109].

Pyroptosis is an inflammatory form of cell death (Figure 2). TLRs and cytokines such as TNF act as a priming signal in pyroptosis, which activate NF-κB to induce the transcription of genes encoding for the inflammasome components and pro-IL-1β [110,111]. The presence of secondary signals such as DAMPs, potassium efflux, nigericin, or ATP then activates the formation of complexes, known as inflammasomes, which trigger pyroptosis and subsequently the release of proinflammatory factors like IL-1β [112,113,114]. Interestingly, DAMPs released during necrotic cell death such as high mobility group protein box 1 (HMGB1), a highly conserved nuclear protein, can also activate TLR2, 4, and 9 [115,116]. In the skin samples of patients with hidradenitis suppurativa (HS), a debilitating skin disease with limited therapeutic options [117], caspase-1 activation was observed in association with the heightened expression of *NLRP3*, *IL1B*, and *IL18* [118]. In HS pathogenesis, the rupture of hair epidermal cysts releases endogenous DAMPs such as high-molecular-weight cornified keratin into the dermis [119], which has been shown to activate the NLRP3 inflammasome [120,121], suggesting that DAMPs and pyroptosis may play a role in exacerbating inflammation in HS.

Necroptosis is another form of programmed cell death (Figure 2). The stimulation of poly(I:C) or TLR4 to FADD-deprived Jurkat cells or mouse macrophages in the presence of caspase inhibitors has been shown to activate RIPK3-mediated necroptosis [122,123]. TLR3 engagement leads to the recruitment of TRIF and interaction with RIPK3 through RHIM domain, promoting the generation of reactive oxygen species (ROS) and necroptosis [123]. In human keratinocyte cell line, HaCaT, TLR3 agonist stimulation sensitised the cells towards RIPK1- and RIPK3-dependent necroptosis in the absence of cIAPs and caspases [124]. In mice injected with poly(I:C) or LPS with zVAD-fmk (pan-caspase inhibitor) led to necroptosis in macrophages and the increased production of IL-6, TNF, MCP-1, and IFN-γ [123]. When RIPK3-deficient mice or dominant negative mutant *Trif*^LPS2/LPS2^ were injected with poly(I:C) or LPS with zVAD-fmk, the death of macrophages was rescued [123]. This study suggests that RIPK3 and TRIF are involved in TLR-mediated necroptosis. It has also been suggested that DAMPs such as HMGB1 may be released during TLR4-mediated necroptosis as the TLR4 signalling pathway contributes to IRAK4-dependent HMGB1 secretion in macrophages [125]. Lastly, it has been shown that TLR3 activation through poly(I:C) is susceptible to the induction of cell death in HaCaT human keratinocytes, and this process requires the activation of caspase-3 by TLR3 and its adaptor, TRIF [126]. TLR4 and MyD88 have also been shown to facilitate UV irradiation-induced cell death in murine macrophages [127].

TLRs are critical components in innate immunity and play a central role in pathogen recognition during infections and maintaining homeostasis. A genetic screening study on 110 healthy human subjects showed that individuals with the TLR2 gene polymorphism (substitution of arginine to glutamine at gene position 753 [Arg753Gln]) had an increased risk of developing staphylococcal infection [128]. Moreover, atopic dermatitis patients with a *TLR2* Arg753Gln gene polymorphism showed an increase in IL-6 and IL-12 levels as compared to patients with no mutation in the *TLR2* gene upon TLR2 agonist stimulation of the monocytes [129]. In a mouse model of the *Staphylococcus aureus* (*S. aureus*) infection, the TLR2- and MyD88-deficient mice were highly susceptible, and the MyD88-deficient mice failed to recruit neutrophils to the infection site [130]. Therefore, TLR2 plays an important role in the pathogenesis of atopic dermatitis and may be involved in enhanced *S. aureus* skin infection [129].

Psoriasis is a well-characterised inflammatory skin disease with keratinocyte hyperproliferation and massive immune cell accumulation. T cells, innate immune cells, and keratinocytes are major contributors to the pathogenesis of psoriasis [131,132,133,134]. In psoriatic skin lesions, heat shock proteins were found to be overexpressed, which stimulated TLR4 on antigen-presenting cells such as the LCs [135]. TLR1 and TLR2 were elevated in the suprabasal keratinocyte layer in non-lesional and lesional psoriatic skin as compared to normal human skin, while TLR5 was downregulated in the basal keratinocyte layer of lesional psoriatic skin as compared to non-lesional psoriatic skin, suggesting that TLRs may play a role in the pathogenesis of psoriasis [89]. Similarly, other skin diseases such as HS have also been associated with the differential expression of TLRs [136,137,138]. In a mouse model of psoriasis, the inhibition of MyD88 in myeloid cells led to the amelioration of the skin phenotype, suggesting a critical role of TLR-mediating signalling during skin inflammation [139].

In several mouse models, which are driven by cell death, the importance of TLR and IL-1 signalling has been demonstrated, highlighting the crosstalk between cell death and TLRs in the disease context. In *Sharpin^cpdm^* mice, in which inflammation is driven by cell death [22,23,60], the deficiency of MyD88 [64] or TLR3 [63] ameliorated skin lesion development. In RIPK1^E-KO^ mice, in which the skin lesions are driven by necroptosis, the deficiency of TRIF mildly ameliorated inflammation [42]. In OTULIN^E-KO^ mice, where skin lesion development is driven by caspase-8-mediated cell death and necroptosis, the deficiency of *Myd88* or injection with Anakinra, an IL-1 receptor inhibitor, strongly protected the development of skin lesions [66,67]. Single-cell RNA sequencing also showed an upregulation of *Il1b* and the genes involved in pyroptosis, such as *Casp1*, *Pycard*, and *Nlrp3*, in the lesional skin of OTULIN^E-KO^ mice [66], which suggests that pyroptosis may be implicated in the pathogenesis.

PTPN6 encoding tyrosine-protein phosphatase non-receptor type 6, which is also known as Src homology 2 (SH2) domain-containing cytosolic phosphatase 1 (SHP-1), is a key component controlling inflammation and cell death [140]. Several different mutations in the mouse *Ptpn6* gene have been associated with neutrophilic dermatoses and autoimmune diseases [141,142]. For instance, functionally inactive PTPN6 (*Ptpn6^me^*^/*me*^ mice) caused the mice to develop patchy hair loss and pigment in the skin before succumbing at 2–3 weeks of age [143,144,145,146,147]. Additionally, the insertion of the B2 element into exon 6 of mouse *Ptpn6* (*Ptpn6^meB^*^2/*meB*2^) also led to the skin inflammation of the paws at 3–5 weeks of age [148]. Interestingly, mice that are homozygous recessive for the missense Y208N spontaneous inflammation (*spin*) in the carboxy terminus of *Ptpn6* gene (*Ptpn6^spin^* mice) develop spontaneous skin lesions, chronic footpad swelling, and purulent inflammation at 8–16 weeks of age [149]. Neutrophils are the main driver of cutaneous inflammation in PTPN6-deficient mice [150].

The deletion of IL-1α, but not IL-1β, alleviates the *Ptpn6^spin^*-mediated skin phenotype; this is not mediated through NLRP3, as crossing these mice with the *Nlrp3*^−/−^ or *Casp1*^−/−^ did not rescue the skin phenotype [151]. However, the pharmacological inhibition of RIPK1 through necrostatin-1 and the genetic blockade of RIPK1, but not RIPK3 deletion, protected *Ptpn6^spin^* mice against wound-induced inflammation [151]. PTPN6 was found to inhibit the Syk-dependent MyD88 phosphorylation and engagement of RIPK1, TAK1, and apoptosis signal-regulating kinase (ASK) to prevent IL-1α signalling; hence, the absence of *Ptpn6* led to inflammatory skin disease development [152,153]. PTPN6 deficiency in polymorphonuclear neutrophils (PMNs) *Ptpn6^ΔPMN^* also led to the inflammatory skin phenotype, which was prevented by the combined deletion of *Casp8^ΔPMN^* and either *Ripk3*^−/−^ or *Mlkl*^−/−^, suggesting that PTPN6 maintains RIPK1’s function to prevent caspase-8 and RIPK3–MLKL-dependent cell death. PTPN6 is also a negative regulator of p38 MAPK, regulating TNF and IL-1α/β expression [154]. Additionally, the ablation of CARD9 in *Ptpn6^spin^* mice led to the downregulation of MAPK and NF-κB signalling, which dampened inflammation [155]. Therefore, the *Ptpn6* murine model provides insights into the mechanisms controlling neutrophilic dermatosis. Clinical findings of these mice closely resemble those observed in human neutrophilic dermatoses, such as Sweet’s syndrome and pyoderma gangrenosum, opening therapeutic opportunities for human skin diseases [156,157,158].

These findings suggest the pro-inflammatory roles of TLRs either as an upstream regulator of inflammation or by acting as PAMP/DAMP receptors during stress. However, more investigation is needed to dissect the inflammation and cell death axes in the context of skin inflammation.

### 3.2. NOD-like Receptors (NLRs)

NOD-like receptors (NLRs) are present in the cytosols of immune and non-immune cells and sense intracellular DAMPs and PAMPs. NLRs are divided into five subfamilies based on different domains at the N-terminus. Here, we will focus on the NOD-like receptor CARD domain-containing (NLRC) family and the NOD-like receptor pyrin domain-containing (NLRP) family [159,160,161]. NLRC contains an N-terminal caspase activation and recruitment domain (CARD), a centrally located nucleotide-binding and oligomerisation domain (NACHT), and carboxy-terminal leucine-rich repeats (LRRs). The NLRPs contain a pyrin domain (PYD) at the N-terminus, the NACHT domain, and LRR domain at the C terminus [162]. Stimulation of NLRs drives the initiation of NF-κB and MAPK signalling pathways and subsequently promotes the expression of pro-inflammatory cytokines and chemokines that recruit immune cells, as well as the activation of inflammasomes that lead to programmed cell death known as pyroptosis (Figure 2) [159,160,161,163].

#### 3.2.1. NLRC Subfamily

The NLRC subfamily is classified based on the presence of the CARD domain at the N-terminus. Amongst the NLRC family, NOD1, NOD2, and NLRC5 are expressed in keratinocytes and immune cells in the skin [163,164,165]. NOD1 and NLRC5 have one CARD domain, while NOD2 has two in tandem. Both NOD1 and NOD2 bind to peptidoglycans. NOD1 recognises γ-D-glutamyl-mesodiaminopimelic acid (iE-DAP), while NOD2 senses muramyl dipeptide (MDP) and single-stranded RNA from viruses (Figure 2) [166,167,168,169,170,171,172,173,174,175]. NLRC5 plays a regulatory role in the innate immune response [176].

Upon ligand recognition, NOD1 and NOD2 recruit RIPK2 via the CARD domain, which leads to the autophosphorylation and ubiquitination of RIPK2 [160,161,163,169,177,178,179,180]. This is further targeted by an X-linked inhibitor of apoptosis (XIAP) and other E3 ligases for non-degradative polyubiquitination, and this process further recruits TAK1 and IKK kinases to initiate the MAPK and NF-κB signalling pathway [169,177,178,179,180]. Through knockdown studies in human keratinocytes, it has been shown that NOD1 mediates *Pseudomonas aeruginosa*-induced CXCL8 secretion [181]. Moreover, the stimulation of primary keratinocytes with MDP results in the release of antimicrobial peptide human β-defensin-2 [182]. Furthermore, the knockdown of NOD2 in keratinocytes reduces *S. aureus*-induced IL-17C expression [183]. These suggest that NOD1 and NOD2 play an important role in the induction of cytokines and chemokines in keratinocytes and help in mounting immune responses against infections [184].

NLRC5 is expressed in keratinocytes and other immune cell types such as lymphocytes and macrophages [176,185]. IFN-γ can also induce NLRC5 and regulate major histocompatibility complex (MHC I) expression in keratinocytes [165,186]. In keloid fibroblasts, silencing NLRC5 inhibits extracellular matrix expression by inhibiting the transforming growth factor-beta1/Smad signalling pathway [187]. NLRC5 has also been shown to negatively regulate NF-κB activation, type I IFN, and inflammatory cytokine production [188]. Lastly, cytomegalovirus-infected human fibroblast showed the upregulation of NLRC5 and the production of IFN-γ-mediated by the Janus kinase-signal transducer and activator of transcription (JAK/STAT) signalling pathway, suggesting that NLRC5 contributes to the antiviral defence response in the skin [189]. Nonetheless, the role of NLRC5 in skin homeostasis and immunity is still unclear.

#### 3.2.2. Nucleotide-Binding Oligomerisation Domain, Leucine-Rich Repeat, and Pyrin Domain-Containing Protein (NLRP)

The NLRP subfamily is classified based on the presence of the pyrin domain at the N-terminus, which allows for the recruitment of the inflammasome-activating scaffold protein, the apoptosis-associated speck-like protein containing a CARD (ASC) [190,191]. NLRP1, 3, and 10 are expressed in human skin [192,193]. Inflammasome activation facilitates the cleavage of caspase-1, pyroptotic cell death, and the processing and release of IL-1β and IL-18 [190,191,194]. Unstimulated human keratinocytes cultured in vitro have been shown to constitutively express pro-*IL1B* and *IL18*, suggesting that keratinocytes do not require priming for their production. [195,196,197,198].

NLRP1 is highly expressed in keratinocytes and is the predominant form of NLRPs in the skin [192,199]. Human NLRP1 is activated by ultraviolet (UV)B radiation [200]. However, UVB irradiation of mouse keratinocytes does not lead to the activation of inflammasomes, indicating that NLRP1 in human and mouse keratinocytes may not be highly conserved [200]. Other activators of human NLRP1 include long dsRNA, bacterial toxin (e.g., nigericin), and viral 3C proteases [201,202,203,204]. Poly(dA:dT), a synthetic dsDNA mimetic, has been shown to induce NLRP1 in human keratinocytes, leading to the activation of canonical inflammasomes (caspase-1, ASC) and IL-1β release [203]. Single-nucleotide polymorphisms in the *NLRP1* gene has also been shown to be associated with several inflammatory skin diseases such as atopic dermatitis and psoriasis vulgaris [205,206,207].

NLRP3 is also expressed in skin and keratinocytes [192,208]. In human keratinocytes, NLRP3 can be activated by various signals such as dsRNA (poly I:C), contact sensitisers, mite allergens, UVB, and oxidative stress. These triggers activate the inflammasome, leading to the cleavage of caspase-1 and pyroptotic cell death [195,196,197,198]. NLRP3 also engages the non-canonical inflammasome, in which caspase-4 and caspase-5 cleave GSDMD to generate pore-forming N-terminal domain GSDMD-N [25,209,210,211,212]. This allows for the release of DAMPs and pro-inflammatory cytokines such as Il-1β and IL-18 [213,214]. Unlike conventionally released cytokines, which employ the endoplasmic reticulum (ER)/ Golgi pathways, IL-1β lacks a signal peptide and is secreted through the GSDMD-N pores [215]. Studies have shown that in UVB-irradiated keratinocytes, caspase-4 was indispensable for pro-IL-1β and pro-IL-18 maturation [216]. Nonetheless, this was dependent on caspase-1 expression as the knockdown of caspase-1 reduced the caspase-4-mediated unconventional secretion of matured IL-1β [216].

In *Sharpin^cpdm^* mice, both canonical and non-canonical NLRP3 inflammasome were implicated [217]. *Sharpin^cpdm^* bone marrow-derived macrophages (BMDMs) primed with LPS (TLR4 agonist) and Pam3CSK4 (TLR2 agonist) and activated with ATP showed decreased caspase-1 and IL-1β compared to the wildtype BMDMs [217]. *Citrobacter rodentium* activates the non-canonical NLRP3 inflammasome through caspase-11 [210]. *Sharpin^cpdm^* BMDMs infected with *C. rodentium* showed reduced caspase-11, caspase-1, IL-1β, and IL-18 production compared to the wildtype BMDMs, showing that SHARPIN is essential for non-canonical NLRP3 inflammasome activation [217]. *Sharpin^cpdm^* mice crossed with mice lacking interleukin-1β converting enzyme (*Ice*^−/−^ mice), which are deficient in both caspase-1 and caspase-11, showed a delayed onset and alleviation of skin inflammation [218]. Additionally, reduced cleaved caspase-3/terminal deoxynucleotidyl transferase dUTP nick end labelling (TUNEL) staining and the decreased expression of both apoptosis and necroptosis effector proteins were observed in the *Sharpin^cpdm^; Ice*^−/−^ mice [218].

It has also been shown that the LUBAC complex, particularly HOIL-1L, is necessary for the activation of the NLRP3 inflammasome, as HOIL-1L^−/−^ mice showed a reduction in IL-1β secretion upon NLRP3 stimulation and survived the lethal inflammation induced by LPS in vivo [219]. Interestingly, patients deficient in HOIL-1L were also highly susceptible to pyogenic bacterial infections [220], which may suggest that NLRP3 inflammasome assembly may be defective in these patients [219]. Overall, these studies suggest that linear ubiquitination is essential for NLRP3 inflammasome activation and governs the pathogenesis of *Sharpin^cpdm^* mice [219]. In *Sharpin^cpdm^* mice, systemic immune cells dysregulation such as the elevation of neutrophils and T cell activation precedes dermatitis [62]. When the *Sharpin^cpdm^* mice were crossed with mice deficient in the IL-1 receptor (*Il1r*^−/−^), which mediates signals from both IL-1α and IL-1β, the absence of IL-1R delayed the progression of dermatitis in the *Sharpin^cpdm^* mice [62]. Further investigation on *Sharpin^cpdm^ Il1a*^−/−^ mice and *Sharpin^cpdm^ Il1b*^−/−^ revealed that IL-1β, but not IL-1α, delayed the onset of dermatitis at the median age of 60 days as compared to *Sharpin^cpdm^* mice at the median age of 42.5 days [62]. Clinically, NLRP3 has also been found to be associated with inflammatory skin diseases such as psoriasis, atopic dermatitis, urticaria, cryopyrin-associated periodic syndrome (CAPS), and vitiligo [194,205,221,222,223].

NLRP10 is another highly expressed NLR in the skin and keratinocytes, and it can be activated upon mitochondrial damage and cellular stress [193,224,225]. NLRP10 lacks the C-terminus leucine-rich repeat (LRR) domain, which is responsible for the recognition of DAMPs and PAMPs; therefore, it differs from other NLRPs [226]. NLRP10 has been shown to bind to the A20-binding inhibitor of NF-κB (ABIN1), a negative regulator of NF-κB via the NLRP10 NATCH domain [227]. Additionally, the pyrin domain of NLRP10 inhibits ASC-mediated NF-κB activation, cell death, and caspase-1-mediated IL-1β maturation [224,228]. In genome-wide association studies, NLRP10 has been linked to several inflammatory skin diseases, including atopic dermatitis and contact hypersensitivity [229,230,231]. The role of NLRP10 has also been shown in the contact hypersensitivity response in mice, where the keratinocyte-specific knockout of NLRP10 resulted in less inflammation [231]. NLRP10 regulates the innate immune response by modulating the p38 and NF-κB signalling pathways in the dermal fibroblast and epithelial cells upon infection with *Shigella flexneri* [193]. This effect was dependent on interactions with NOD1 and its binding partners, RIPK2, TAK1, and NEMO [193]. In a cutaneous infection with the West Nile virus, LCs migrate to the lymph node, and the neutralisation with anti-IL1β, but not anti-TNF, inhibited this migration [232]. The mechanism of IL-1β-dependent LC migration in a cutaneous West Nile virus infection is unclear; however, the involvement of inflammasomes can be speculated. These studies highlight the crucial roles of NLRs in skin homeostasis, inflammation, and infection, and further research is needed to explore their exploitation as therapeutic targets.

### 3.3. Nucleic Acid Sensors

#### 3.3.1. Absence in Melanoma 2 (AIM2)

The absence in melanoma 2 (AIM2) is a cytoplasmic DNA sensor which recognises dsDNA and is best known for its defence role against bacteria and viruses [233,234,235,236,237,238]. The binding of cytosolic DNA to AIM2 leads to the self-assembly of inflammasome adaptor protein ASC [233,236,237]. This allows for the recruitment of pro-caspase-1 to the inflammasome, leading to the processing of caspase-1 and the maturation of pro-IL-1β and pro-IL-18 [233,237,239]. However, AIM2 lacks sequence specificity in recognising DNA; therefore, it can also detect endogenous self-nucleic acid that could be released during cellular stress or cell death [238,240]. Consequently, this can have significant implications for autoimmune diseases as the recognition of self-DNA would activate autoreactive immune cells, break self-tolerance, and amplify autoimmune response and tissue damage.

Interestingly, AIM2 has been shown to crosstalk with the apoptosis pathway during infection. The AIM2/ASC complex activated caspase-8-mediated cell death in caspase-1 knockout macrophages upon infection with intracellular bacteria, *Francisella novicida* [241]. Moreover, the inhibition of caspase-8 or caspase-9 or the overexpression of Bcl-2 or Bcl-X_L_ rescued caspase-8-mediated cell death in caspase-1 knockout macrophages, indicating that the mitochondrial intrinsic pathway is involved in *F. novicida* infection [241]. In a separate study, the electroporation of CT DNA (ds homopolymer of Poly(dA:dT) in bone marrow-derived macrophages from caspase-1 knockout mice led to caspase-1-dependent pyroptosis and caspase-1-independent cell death mediated by ASC [242]. Additionally, both AIM2 and NLRP3 inflammasomes were shown to activate caspase-8 and initiate caspase-8-dependent cell death and pyroptosis [242]. Moreover, in primary human monocyte-derived macrophages, *Toxoplasma* infection induced AIM2-dependent apoptosis [243]. Infection of AIM2-knockdown dermal fibroblast with Chikungunya or the West Nile virus led to a decrease in IL-1β production [244]. AIM2 expression was also elevated in primary human skin fibroblasts infected with the Zika virus, which resulted in IL-1β production [245]. These studies emphasise the adaptability of the AIM2 inflammasome in eliciting diverse immune responses depending on the circumstances.

AIM2 has been implicated in inflammatory skin diseases such as psoriasis, atopic and contact dermatitis [205,240,246,247,248,249], and viral skin infections [250]. For example, herpes simplex virus type 1 (HSV-1) activated both AIM2 and NLRP3 inflammasomes in IFN-γ-stimulated human primary keratinocytes [250]. Human keratinocytes primed with IFN-γ and TNF followed by stimulation with Poly(dA:dT) enhanced the release of IL-1β as compared to unprimed keratinocytes [246,250]. The knockdown of *AIM2* in human keratinocytes diminished the IFN-γ-primed enhanced responses and IL-1β release upon HSV-1 infection [250]. Interestingly, the anti-microbial cathelicidin peptide, LL-37, is increased in psoriatic skin and has been proposed to be a physiologic inhibitor of AIM2 inflammasome activation [246]. In this study, LL-37 reduced the AIM2-dependent release of IL-1β in human keratinocytes [246]. In another study, the AIM2 inflammasome was expressed in the entire epidermis of both the healthy and lesional skin of the AD and psoriasis patients [251]. Furthermore, in response to Poly(dA:dT) transfection, healthy human keratinocytes released IL-1β without IFN-γ stimulation [251]. These studies and the existing discrepancies highlight the need for more studies on the role of AIM2 in skin diseases and infection [246,251].

#### 3.3.2. Cyclic GMP-AMP Synthase (cGAS) and Interferon-γ Inducible Protein 16 (IFI16)

cGAS recognises cytosolic dsDNA, and its enzymatic domain catalyses the synthesis of 2′ 3′ cyclic GMP-AMP (cGAMP), a second messenger, which binds and activates the ER membrane protein, stimulator of interferon genes (STING) [252,253,254,255]. cGAMP binding to STING results in confirmational changes that allows for the binding of TBK1 and interferon regulatory factor 3 (IRF3) [252,253,254,255]. TBK1 phosphorylates IRF3, which allows for its dimerisation and nuclear translocation to induce type I interferon and interferon-stimulated genes (ISGs) [252,253,254,255].

STING also engages the NF-κB pathway and induces the expression of inflammatory cytokines such as TNF, IL-1β, and IL-6 [252,253,254,255]. TBK1 has been shown to be dispensable for NF-κB activation, and the TBK1 homolog, IKKε, plays a redundant role with TBK1 to initiate STING-induced NF-κB activation [256]. NF-κB activation has been demonstrated to promote STING signalling. The deletion of p65 in THP-1, a human monocytic cell line, completely blocks the activation of NF-κB by TLRs, interleukin-1 receptors, TNFRs, protein kinase C, and growth factor receptors, thus preventing the enhancement of STING signalling [257]. Moreover, NF-κB activation prevents STING degradation, enabling prolonged and robust STING signalling [257]. The cGAS-STING pathway is associated with ER stress and the NLRP3 pathway. HEK293T cells transfected with cGAS and STING upregulate the markers of ER stress and unfolded protein response [258,259]. Moreover, cGAS-STING has been shown to interact with Ca^2+^ sensor stromal interaction molecule 1 (STIM1) [258,260]. After HSV-1 infection or cytosolic DNA stimulation, STING binds to NLRP3 and promotes the activation of the NLRP3 inflammasome through NLRP3 localisation in the ER [261].

Interferon-γ-inducible protein 16 (IFI16) and cGAS-STING signalling pathway play a critical role in keratinocyte inflammatory responses [262]. IFI16 is expressed in human cells, and mice possess its orthologue, p204. IFI16 shuttles between the nucleus and the cytosol [236]. Under a steady state, it is predominantly localised in the nucleus but is also a well-known cytosolic DNA sensor [262,263]. During a vaccinia virus infection in the human keratinocyte cell line, HaCaT, the expression of IFI16 was detected in both the nucleus and cytoplasm, which led to the elevated expression of *IFNB*, *ISG56*, and *IL6* mRNA [263]. IFI16 interaction with cGAS was required for the DNA-induced activation of STING and IRF3 in HaCaT cells [263]. The mouse orthologue of IFI16, p204, has been shown to interact with STING and promote IRF3 and NF-κB activation in mouse macrophages [264]. Additionally, UVB-induced DNA damage in HaCaT cells activated the cGAS-STING and NF-κB and IRF3-IFN I pathways, leading to apoptosis of the cells [265].

In recent years, CRISPR/Cas9 technology has been suggested for the treatment of congenital skin disorders. However, it has been proposed that the human keratinocyte cell line (N/TERT) exhibits low transfection efficiency with CRISPR/Cas9 transfection due to poor plasmid uptake and rapid degradation [266]. The study showed that in recognition of the CRISPR plasmid, STING was activated, triggering a response specific to IFN-κ, a predominant type I IFN expressed by basal keratinocytes [266]. The production of IFN-κ directly affected cytidine deaminase APOBEC3G expression, which led to the degradation of the intracellular CRISPR plasmid thus reducing transfection efficiency in the keratinocytes [266]. Nevertheless, regardless of the target gene, suppressed *IFNK* and *ISG* mRNA expression was observed in the successfully CRISPR/Cas9-generated KO keratinocytes [266]. Moreover, *IFNK* deficiency in the keratinocytes interrupted the IFN autocrine signalling, which led to the improvement in CRISPR/Cas9 transfection efficiency [266]. This study highlighted the mechanism by which STING plays a role in mediating CRISPR/Cas9 transfection resistance in keratinocytes [266].

STING and IFI16 have been associated with skin diseases such as psoriasis [267,268,269]. The lesional skin of psoriasis patients has shown increased mRNA expression of gene encoding for STING (*TMEM173*) and its downstream targets, such as *TBK1*, *NFKB1*, *NFKB2*, and *IL6* [267]. In a mouse model of imiquimod-induced psoriasis-like inflammation, the application of H-151, a STING antagonist, alleviated the inflammatory skin phenotype [267], while in *Tmem173*^gt^ mice, the imiquimod-induced inflammation was attenuated [269]. Additionally, mice with selective loss of STING in the dendritic cells (*CD11c*-*Cre*-*Sting*^−/−^) showed an attenuated skin phenotype [268]. Mechanistically, in vitro studies have shown that H-151 inhibits STING/NF-κB signalling in THP-1 monocytic cells [267]. In another study, cytosolic DNA synergised with TNF and induced the expression of *Il1b* mRNA in keratinocytes through the STING-dependent NF-κB pathway [269]. Moreover, in vitro stimulation with cytosolic DNA and TNF showed the reduced expression of *Ccl20* and *Cxcl10*, chemokines associated with psoriasis, in STING-deficient keratinocytes [269].

#### 3.3.3. Retinoic Acid-Inducible Gene (RIG)-I-like Receptors (RLR), RIG-I, and Melanoma Differentiation-Associated Protein 5 (MDA5)

RIG-I and MDA5 are protein sensors of viral dsRNA [270,271]. Upon binding to dsRNA, RIG-I and MDA5 interact with the mitochondrial antiviral signalling protein (MAVS) [272,273,274]. This allows for the formation of molecular complexes, which eventually leads to an interaction with TBK1 and IKKϵ. TBK1 and IKKϵ then phosphorylate and translocate the transcription factors IRF3 and IRF7 into the nucleus, where they activate the type I interferon genes, IFNα and IFNβ [270,275,276,277]. In addition, both RIG-I and MDA5 activate the NF-κB pathway in a respiratory syncytial virus (RSV) infection, whereby RIG-I acts upstream of the canonical NF-κB pathway and mediates the translocation of the p50 and p65 NF-κB subunits into the nucleus [278]. The mechanisms of how MDA5 influences the NF-κB pathway are unclear, though it is believed that it acts independently of IκBα [279]. Both RIG-I and MDA5 induce the NF-κB-mediated production of IL-6 and pro-IL-1β through the interaction of CARD9 and BCL10 [280,281].

RIG-I is constitutively expressed in the cytosol of oral keratinocytes (RT7) and HaCaT cells [282,283]. Moreover, the transfection of Poly(I:C) induced the mRNA expression of *IFNB* and *CXCL10* via the phosphorylation of IRF3 and STAT1 in the RT7 cells [282]. In a separate study, the incubation of RT7 cells with LL-37 increased necrotic cell supernatant-induced *CXCL10* mRNA expression, in which its expression was significantly reduced upon dsRNA cleavage with RNase III digestion [284]. The LL37- and Poly(I:C)-induced level of *CXCL10* mRNA was attenuated by the inhibition of the NF-κB pathway using the inhibitor, Bay11-7082, suggesting that NF-κB signalling regulates the viral immune response in oral keratinocytes [284]. These studies emphasise the role of RIG-I in the detection of dsRNA and its coordination with NF-κB in the modulation of the immune response to a viral infection in the oral keratinocytes. In HaCaT cells, IFN-γ or TNF stimulation increased RIG-I protein expression as compared to untreated cells [283]. Furthermore, the expression of *RIGI*, *IFIH1* (encodes for MDA5), *IRF3*, *IRF7*, and IRF-dependent antiviral genes in Chikungunya virus-challenged primary human keratinocytes were found to be elevated as compared to those with the mock treatment, indicating that the innate immune response was activated in keratinocytes during an infection [285].

In mice with streptozotocin-induced diabetes and wound samples of diabetic feet, where wound healing was impaired, decreased RIG-I expression was observed [286]. NF-κB signalling was further suggested to facilitate RIG-I function, promoting keratinocyte proliferation and skin wound repair through tissue inhibitor of metalloproteinase-1 (TIMP-1). This suggests that RIG-I may be a biomarker and therapeutic target for skin injuries [286]. RIG-I protein expression was also highly upregulated in the spinous and basal keratinocyte skin layer of psoriasis vulgaris samples as compared to healthy skin, suggesting that RIG-I may be involved in psoriasis pathogenesis [283,287]. Indeed, RIG-I has been found to induce IL-23 production by myeloid dendritic cells [287].

In human patients with progressive vitiligo, the MDA5 level was found to be elevated as compared to the healthy control [288]. Moreover, the study found an association between cytomegalovirus infection and progressive vitiligo, in which MDA5 plays a key role in promoting the secretion of chemokines, CXCL10 and CXCL16, in the epidermis and the infiltration of CD8^+^ T cells in the skin tissues [289]. The mechanistic study showed that poly(I:C) stimulation of normal human epidermal keratinocyte (NHK) cells also led to an increase in IFN-β secretion, mediated through the MDA5-MAVS-NF-κB/IRF3 signalling pathway [289]. This consequently led to the production of CXCL16 mediated by the MDA5-IRF3 pathway and CXCL10 secretion via the JAK1/STAT1 pathway [289]. Lastly, single-cell RNA sequencing data showed that the RIG-I pathway genes were elevated in the lesional skin of systemic lupus erythematosus (SLE) and discoid lupus erythematosus (DLE) patients [290,291,292,293,294]. In addition, UVB irradiation of HaCaT cells led to elevated levels of human endogenous retrovirus dsRNA transcription and activated the RIG-I/MDA5/ IRF7 pathway, leading to ISG expression, which further promoted UVB-induced cell death and the inhibition of HaCaT cells proliferation [295].

#### 3.3.4. Z-DNA Binding Protein (ZBP1)

Z-DNA binding protein (ZBP1) is an innate immune sensor that recognises the Z-form of double-stranded nucleic acids and adopts a left-handed double-helical structure [296]. ZBP1 contains two N-terminal Zα domains that bind to Z-form nucleic acid and has three C-terminal receptor-interacting protein (RIP) homotypic interaction motifs (RHIMs) that allow for interactions with other RHIM-containing proteins such as RIPK1, RIPK3, and TRIF [297]. ZBP1 is involved in inflammation and different forms of programmed cell death, apoptosis, necroptosis, and PANoptosis [298,299,300,301]. PANoptosis is an inflammatory form of programmed cell death regulated by PANoptosomes, which serve as a central signalling hub integrating different programmed cell death such as apoptosis, necroptosis, and pyroptosis [5,299,302]. This process is orchestrated by caspases and RIPKs through the assembly of PANoptosomes, which are multiprotein complexes formed in response to the recognition of PAMPs and DAMPs by cytosolic innate immune sensors [5,299,302].

The sensing of Z-nucleic acid activates ZBP1 and enables the formation of a complex with RIPK3 via an RHIM-mediated interaction [303]. This interaction promotes the RIPK3-mediated phosphorylation of MLKL to execute necroptosis. Moreover, the ZBP1, RIPK3, and caspase-8 assembly triggers NLRP3 inflammasome activation to facilitate pyroptosis [304]. ZBP1 has also been shown to act as an adaptor for the multiprotein assembly facilitating PANoptosis [305]. Within TLR signalling, the assembly of ZBP1, RIPK1, RIPK3, and TRIF also facilitates cell death [306]. In mice deficient in caspase-8 and TNFR1, ZBP1 and TRIF are capable of initiating necroptosis [307]. In the absence of RIPK3, ZBP1 can also induce apoptosis by interacting with RIPK1 and activating caspase-8-dependent apoptosis, leading to a non-lytic form of cell death [43]. Lastly, when RIPK1 is absent in murine embryonic fibroblasts, ZBP1 can drive RIPK3-mediated cell death induced by IFNs [308]. Deletion of *Zbp1* or the core IFN signalling components prolonged the survival of *Ripk1*^−/−^ mice [308].

The role of ZBP1 in viral infections, such as influenza and cytomegalovirus, has been well investigated [304,309,310,311]. For instance, in murine cytomegalovirus infection, ZBP1 interacts with RIPK3 to mediate virus-induced necrosis [309]. However, viruses possess proteins that mimic the host’s cell death proteins [311]. For instance, MCMV M45 contains a viral inhibitor of RIP activation (vIRA), which is a viral RHIM that mimics host RHIM. The interruption of ZBP1-RIPK3 interaction via vIRA eventually inhibits necroptosis [309,311]. HSV-1 and HSV-2 encode for ICP6 and ICP10, respectively. These viral encoded proteins contain viral RHIMs that inhibit RIPK3-mediated necroptosis in human cells [312,313]. Additionally, the varicella zoster virus that causes chickenpox and shingles also produces open reading frame 20, a capsid triplex protein that contains RHIM [314]. The interaction of RHIM with ZBP1 sequesters ZBP1 into decoy amyloid assemblies and prevents ZBP1-driven apoptosis during a varicella zoster virus infection of the human colorectal adenocarcinoma cell line, HT-29 [314]. Other viruses, such as the vaccinia virus also produce vaccinia virus protein E3 to compete for Z-form RNA through an N-terminal Zα domain thus blocking ZBP1/RIPK3/MLKL-dependent necroptosis during infection [315].

ZBP1 is a central player in the influenza A host defence. *Zbp1*^−/−^ and wildtype mice exhibit different levels of susceptibility towards influenza A infection. Moreover, the different domains of ZBP1 have also been shown to control in vitro cell death [303,304,316,317,318,319]. The ZBP1 Zα domain has also been shown to be a key PANoptosome sensor and interacts with RIPK3, caspase-8, caspase-6, and RIPK1 to form the ZBP-1 PANoptosome complex and activate PANoptosis [305,320,321]. In recent years, studies have shown that ZBP1 is a key mediator of PANoptosis and drives NLRP3-inflammasome activation during an influenza infection, which subsequently mediates cytokine release [299,302]. A deeper understanding of the molecular mechanisms regulating ZBP1 is still required to explore ZBP1’s function in managing viral skin infections.

In addition to its role in viral sensing, ZBP1 also plays an important role in autoinflammation [301]. During skin homeostasis, the RHIM domain of RIPK1 prevented ZBP1-mediated necroptosis in mice [322]. In the full-body RIPK1 knockout mice (*Ripk1*^−/−^) or the mice expressing mutant RIPK1, the mutation of the RHIM residues IQIG to AAAA (*Ripk1^mRHIM^*^/*mRHIM*^) led to perinatal lethality due to RIPK3-MLKL-mediated necroptosis [319,322,323]. Mice lacking epidermal RIPK1 (RIPK1^E-KO^) or the RIPK1 RHIM domain (RIPK1^mRHIM/E-KO^) developed progressive inflammatory skin lesions, which were triggered by RIPK3-ZBP1-MLKL-dependent necroptosis [42,322]. The skin phenotype of RIPK1^E-KO^ mice was partially dependent on TNFR1 [42]. In the subsequent studies, crossing RIPK1^E-KO^ mice with the knock-in mice expressing ZBP1 with a deletion (*Zbp1^∆Zα^*^/*∆Zα*^) or mutation of both Zα domains (*Zbp1^mZα^*^1–2/*mZα*1–2^) or Zα2 alone (*Zbp1^mZα^*^2/*mZα*2^), substituting key amino acids (Zα1:N46D, Y50A; Zα2: N122D, Y126A) that are essential for Z-NA binding, prevented severe skin lesion development until up to 18–42 weeks of age [319]. In another study, the Zα domains of ZBP1 were also shown to trigger the inflammatory skin phenotype in the RIPK1^E-KO^ mice [31]. Additionally, the RIPK1^E-KO^ mice crossed with the *Zbp1^mR^*^1/*mR*1^ mice expressing ZBP1 with four amino acid substitutions disrupting its core RHIM sequence (192IQIG to 192AAAA) developed only a mild lesion by the age of 40 weeks [319]. Furthermore, the treatment of RIPK1^E-KO^ mice with retroviral drugs ameliorated the skin phenotype, suggesting the role of endogenous retroviral elements in skin inflammation [319]. Moreover, the embryonic lethality of *Ripk1^mRHIM^*^/*mRHIM*^ was also dependent on ZBP1 [319]. ZBP-1 also has an autoinhibitory function governed by RHIM-B, RHIM-C, and the C-terminal end of ZBP1 [43]. The truncated C-terminus of mouse ZBP1 (ZBP1ca) which led to the loss of 203 amino acid residues, consisting of the Zα domains and RHIM-A, was shown to be constitutively active [43]. The expression of ZBP1ca triggered keratinocyte cell death and led to the development of inflammation skin lesions, suggesting that ZBP1ca could drive skin inflammation [43]. Moreover, the abrogation of RIPK3-MLKL-dependent necroptosis could only partially rescue the inflammation skin phenotype [43]. Nonetheless, the combination of the inhibition of RIPK3-MLKL-dependent necroptosis and RIPK1-caspase-8-driven cell death could fully prevent skin inflammation mediated by ZBP1ca [43]. Lastly, the inhibition of RIPK1 kinase activity and mutation of the MLKL phosphorylation sites did not prevent caspase-8-mediated inflammation in ZBP1ca^E-het^ mice, suggesting that ZBP1 induces RIPK1 kinase activity independent cell death [43].

In another study, ZBP1-mediated necroptosis in human keratinocytes was dependent on IFNγ secretion, as intervening with IFNγ signalling through JAK inhibition led to the inhibition of cell death [324]. Mice lacking epidermal FADD developed necroptosis-mediated skin inflammation [39]. Neither TNFR1 deficiency nor ZBP1 ablation alone was sufficient to inhibit skin inflammation; however, the double deficiency of ZBP1 and TNFR1 prevented skin inflammation [39,43,322]. These studies show that ZBP1-mediated necroptosis drives TNFR-1-independent skin inflammation in mice lacking epidermal FADD. This suggests that dysregulated ZBP1 signalling may disrupt skin homeostasis and contribute to inflammatory skin diseases. Further studies are required to illustrate the impact of ZBP1 on the inflammatory landscape of human skin diseases.

## 4. Concluding Remarks

In this review, we have outlined the innate immune sensors and cell death pathways, their relationships, and their potential relevance to skin inflammation and infection in mice and humans. Although there is functional redundancy in the recognition of nucleic acids amongst the different innate immune sensors within the keratinocytes and immune cells of the skin, this genetic redundancy enables the host to cope with foreign and host molecules, including pathogen evasions. Moreover, the convergence of multiple downstream signalling pathways could facilitate PAMPs or DAMPs recognition by different innate immune sensors in keratinocytes and induce a robust immune response to maintain skin homeostasis. Nevertheless, innate immune sensors could also be a double-edged sword in host immunity as their dysregulation could lead to imbalanced epithelial–immune cell communication and ultimately result in inflammatory skin diseases. Further studies in mice and humans on the regulation of innate immune sensors and cell death pathways, as well as their cellular interaction in the skin, are required to explore their therapeutic potentials during inflammatory skin conditions.
